# Workers of a drywood termite do not work

**DOI:** 10.1186/1742-9994-4-7

**Published:** 2007-02-22

**Authors:** Judith Korb

**Affiliations:** 1 Biologie I, University of Regensburg, D-93040 Regensburg, Germany

## Abstract

**Background:**

Social insects (ants, bees, wasps and termites) are considered as prime examples of altruism in which individuals (workers) forego their own reproduction to help other individuals reproduce. Such a behaviour is favoured by natural selection because the workers rear close kin and in doing so enhance their inclusive fitness.

**Results:**

Here I show, however, that this does not generally apply to termite workers which are scarcely investigated. In the basal drywood termite *Cryptotermes secundus *the 'workers', which form the large majority of a colony, did not stay to raise relatives. There is no brood caring behaviour and they do not engage in costly help. They are large immature offspring that develop into either winged (dispersing) or unwinged (replacement) reproductives and the probability that they did so was unaffected by the number of brood in the nest as a brood addition experiment showed.

**Conclusion:**

Thus, in contrast to general perception where termite workers are considered equivalent to workers in Hymenoptera, the 'large immatures' of *C. secundus *did not behave as workers that help in raising younger siblings. This apparently is not necessary as the colony lives inside its food. These results, which are likely to be typical for wood-dwelling termites, open the possibility that large complex group living can evolve without altruistic helping and that costly altruistic helping by workers in termites evolved only as a second step.

## Background

One of the most intriguing problems in evolutionary biology is the evolution of cooperation, and in particular of altruism (i.e. helping others at own costs). How can such behaviours evolve under competition-driven Darwinian selection? Social insects in which individuals (workers) forego at least some of their own reproduction to help other individuals reproduce are prime examples to investigate this question. In social insects, worker behaviour is generally associated with costs in direct reproduction [1,2]. Even in species such as paper wasps, in which workers and queens are not morphologically different, workers have lower direct reproduction because they probably cannot do both carry out the risky tasks of foraging and laying eggs (e.g. [3-5]). Such altruistic behaviour is favoured by natural selection because in most social insects, the workers rear close kin and in doing so enhance their inclusive fitness [6]. However, these conclusions are mainly based on social Hymenoptera (ants, some bees, and wasps), while few studies exist on the oldest social insects, the termites, that independently evolved sociality [7,8].

**Figure 1:**
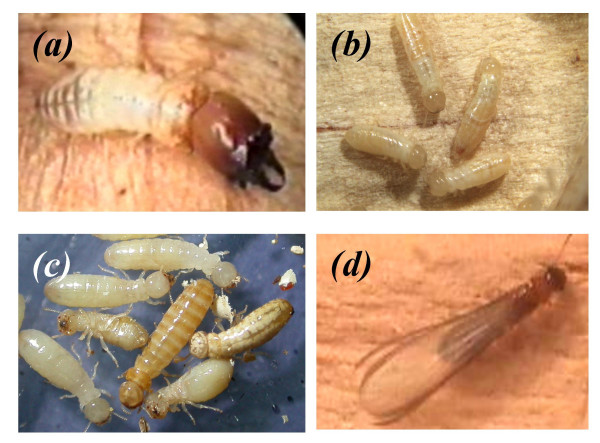
**Castes in *Cryptotermes secundus***. (**A**) A sterile soldier. (**B**) 'Large immatures', generally called helpers or workers as they are assumed to provide help like in other social insects. (**C**) Two neotenic replacement reproductives (brown individuals) together with 'large immatures'. Neotenic replacement reproductives develop from 'large immatures' through a single moult when the reproductives of a colony die. (**D**) Winged sexual (alate). They develop from 'large immatures' through five nymphal instars (individuals with wing buds) and leave the colony to found a new colony.

Wood-dwelling termites, such as *Cryptotermes secundus *(Kalotermitidae) live in a single piece of wood that is both nest and food [9]. This lifestyle is considered the ancestral state in termite evolution [7,10]. It is associated with a flexible development in which the immature individuals of both sexes (normally called workers, pseudergates, or helpers; see [7]; in this study I refer to them as 'large immatures' to separate function from developmental stage) have the possibility of developing into all possible castes, including sterile soldiers and reproductives [7]. In the drywood termite *C. secundus*, a few individuals per nest (1–10; median: 2) are sterile soldiers (Figure 1) and this is undoubtedly a case in which inclusive fitness benefits are involved [11]. The remaining 'large immatures' (individuals above 4^th ^instar in *C. secundus*; [12]), which form the largest group in the colony (c. 95 % of the individuals when excluding small larval instars and eggs), can either stay in the natal colony or can leave by developing into a winged sexual (alate) and found a new colony (Figure 1) [12]. The latter they do, for example, if the amount of food in the nest declines [12]. If they stay in the nest they could gain indirect fitness benefits as workers by raising offspring or/and direct fitness as a neotenic replacement reproductive when the king or the queen of the colony dies (Figure 1).

I investigated the importance of indirect fitness benefits for 'large immatures' who stay as supposed workers at the nest instead of leaving as winged sexuals. First, I tested the hypothesis that 'large immatures' stay in the colony in order to help raise young offspring by experimentally increasing the opportunity to help through boosting the proportion of young individuals in the colony. If these 'large immatures' are actually staying in order to raise young then the presence of young should increase the value of helping and fewer should develop into dispersing reproductives. These 'add young' colonies had roughly 20 % more young instars and eggs than they had originally. They were compared to colonies with an unaltered colony composition (control). To control for handling effect (i.e. addition of individuals *per se*), in a third trial individuals were added, but the age composition was not changed ('add all'). Second, I performed behavioural observations to test the hypothesis that 'large immatures' help in raising young. The results showed that contrary to current perceptions, these supposed termite workers (i.e. the 'large immatures') neither seem to stay in the colony to gain indirect benefits by helping to raise young offspring nor did they help in raising young at all.

## Results

### Do 'large immatures' stay when the number of young offspring is increased?

There was no significant interaction between colony size and experiment showing that the regression slopes of the three treatments ('control', 'add all', 'add young') did not differ significantly (Table 1). In contrast to what we would expect if 'large immatures' stay to gain indirect fitness benefits by raising young, there was no effect of adding young, and the addition of individuals *per se *('add all') also did not have an effect: The number of 'large immatures' leaving the nest did not differ significantly between the three trials, 'control', 'add all', and 'add young' (control: 15.5 ± 10.4; add young: 9.6 ± 4.8; add all: 14.2 ± 6.7; Table 1), while colony size had a significant effect (Table 1). The effect size f was 0.20 and the partial eta^2 ^showed that trial accounted for only 4 % of the total variability in number of 'large immatures' leaving the colony, while 62 % of the variability was explained by colony size.

**Table 1:** Effects of the experimental trials and colony size on the number of dispersing 'large immatures'

**Independent variables**	***F***	***df***	***p***
Trial	0.93	2,20	0.412
Colony size	38.49	1,20	< 0.001
Trial × Colony size	2.52	2,18	0.108

Shown are the results of an ANCOVA analysis of the experimental trials (control, add young, add all) (fixed effects) and colony size (covariate) on the number of dispersing 'large immatures'.

**Figure 2:**
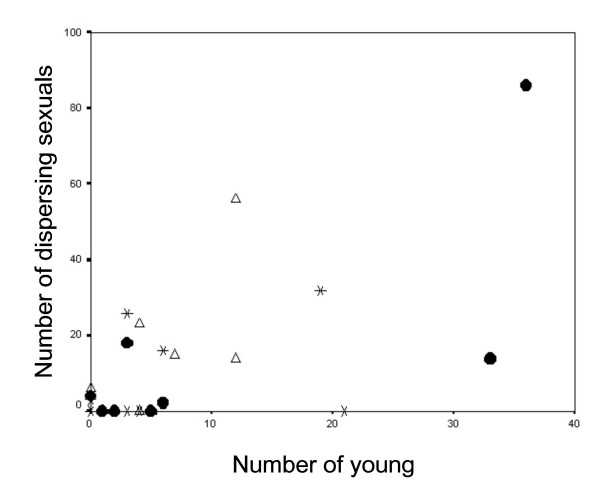
**Number of dispersing individuals in relation to number of young instars present in a colony**. Filled circles: control colonies; asterisks: colonies in which young individuals were added; open triangles: colonies in which individuals were added, but the age composition of the colony was not changed.

The result that the number of dispersing sexuals did not decline when the number of young instars in a colony was increased also cannot be explained by the death of the added young. At the end of the experiment the total number of individuals did not differ between the control and 'add young' colonies (control: 88.7 ± 25.7; add young: 77.6 ± 21.6; t-test: *t*_*14 *_= -0.33, *p *= 0.745) showing that they did survive despite the fact that the 'large immatures' were leaving as winged sexuals (see also Methods). The effect size f was 0.15. There was also no significant negative correlation in any of the trials between the number of 'large immatures' leaving the colony and the number of young instars present in the nest (Pearson's tests: control: *r*^2 ^= 0.072; *F*_*1,8 *_= 0.54; *p *= 0.485; add all: *r*^2 ^= 0.352; *F*_*1,8 *_= 3.79; *p *= 0.092; add young: *r*^2 ^= 0.094; *F*_*1,8 *_= 0.73; *p *= 0.422) (Figure 2) as would have been expected when 'large immatures' stay to raise young offspring. On the contrary, when all trials were combined, the number of individuals leaving the nest increased with the number of young instars present in the nest at that time (Pearson's test: *r*^2 ^= 0.391; *F*_*2,24 *_= 14.11; *p *= 0.001) (Figure 2). These results strongly suggest that indirect benefits derived from helping are not the driving force for 'large immatures' staying at the nest.

### Do 'large immatures' help?

**Figure 3:**
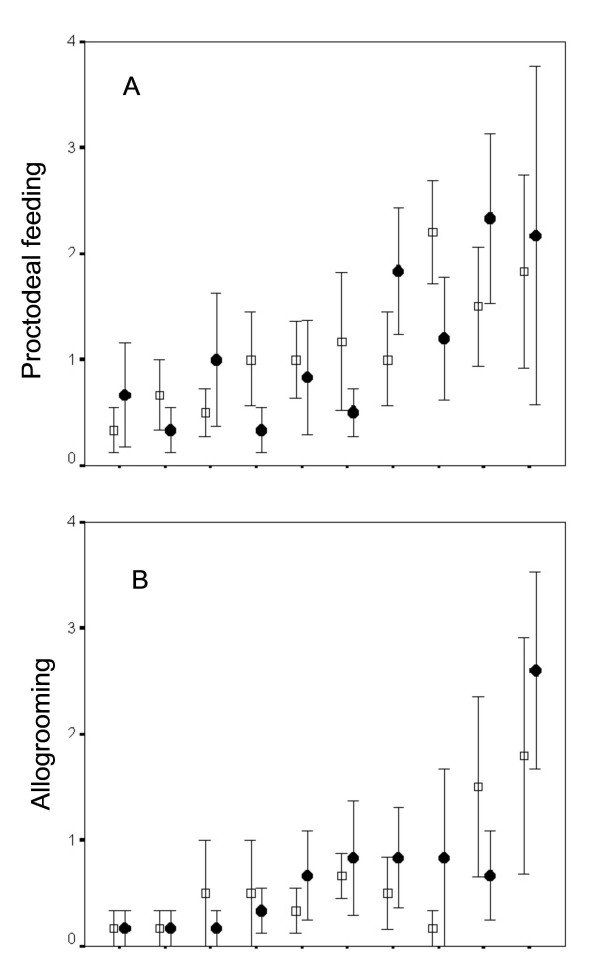
**Mean frequency (± s.e.m) of (A) proctodeal feeding and (B) allogrooming**. Ten individuals from five colonies were each investigated six times. The presented sequence of individuals was sorted by frequencies. Open squares: active behaviour; filled circles: passive behaviour.

Contrary to current perceptions, the supposed *C. secundus *'workers' (i.e. large immatures) did not really help to raise their siblings. In particular, they provided no specialised brood care or defence. Foraging, an important and risky task normally carried out by worker insects, is unnecessary as the colony lives within its food, a piece of wood. The only two behaviours that might be categorised as altruistic helping are proctodeal feeding and allogrooming. However, these behaviours are not costly to the actor. Behavioural observations of 'large immatures' showed that for each individual the frequency of feeding or allogrooming others did not differ from that of being fed or allogroomed (Wilcoxon tests: for each of the 10 individuals: proctodeal feeding: *p *> 0.05, *n *= 6 observation periods; allogrooming: *p *> 0.05, *n *= 6 observation periods; Figure 3). So called 'dependent' larval instars (i.e. ≤ 3^rd ^instar) were not observed to be fed or allogroomed during this experiment nor during any other observation since the project started, covering more than 300 observation hours of more than 600 individuals from more than 40 colonies during all seasons. From the second instar larvae onward (including reproductives) individuals were seen to feed themselves. Eggs and first instar larvae were not cared for, they are not carried around, piled up or licked; but also they did not grow obviously until the next moult, which might suggest that they utilize body reserves. The only individuals that needed to be fed were the soldiers. However, this presents a minor cost as less than 5 % of the individuals (median: 2) were soldiers. On a per capita basis each 'large immature' fed a soldiers less frequently than once per day. Furthermore, it is difficult to classify this feeding of soldiers as truly altruistic as the soldiers are the 'true' altruists that defend the colony and thereby increase directly the survival of the feeding individuals.

## Discussion

Contrary to current perceptions, these supposed termite workers (i.e. the 'large immatures') do not seem to stay in the colony to gain indirect benefits by helping to raise young offspring. They were not less likely to leave the colony as winged sexuals when the potential to help was increased by boosting the proportion of young instars. The effect size was medium and addition of young accounted for only 4 % of the variability in the number of dispersing 'large immatures', while more than 50 % of the variability was explained by colony size. On the contrary, the number of 'large immatures' leaving the colony correlated positively with the number of young instars. Furthermore, despite the dispersal of the 'large immatures' in the 'add young' experiment, the young instars survived. These results are explained by a general lack of helping behaviour directed at young. As in other basal termites [13,14], no brood care occurred. It is not necessary in *C. secundus *because of the termites' hemimetabolous development and the fact that the colony lives inside its food. Thus, in contrast to the holometabolous social Hymenoptera, young are relatively independent and no costly foraging exists. Therefore, exactly those conditions are absent in wood-dwelling termites that Queller and Strassmann [15] identified as the most important factors selecting for the evolution of workers: care of young and food provisioning. In termites, this changes with the transition to non-wood nesting species, in which the nest and the foraging area are separate (so-called 'multiple life type termites'; [9]). Correspondingly, in these termites true workers occur which feed the brood and have a reduced capability to reproduce [7]. Among 'large immatures' in *C. secundus*, all individuals performed all tasks and supposedly altruistic behaviours, i.e. proctodeal feeding and allogrooming, were none costly reciprocal interactions. The interactions among nestmates should therefore not be regarded as costly altruistic helping which supports the conclusion of the experiment that these 'large immatures' do not stay in the colony for kin's sake.

This conclusion is also supported by other features of social life in this species. In particular, reduction to zero relatedness between 'large immatures' and newborn offspring, does not lead to more individuals leaving the nest [16]. Such a reduction in relatedness naturally occurs in about 25 % of all field colonies, namely when colonies that were founded in the same piece of wood fuse during colony expansion and both reproductives of one colony are killed. In such situations the 'large unrelated immatures' do not respond by leaving the nest, although they do adaptively adjust development in other situations, for example, when food availability declines [12,17].

Although these results were derived specifically for *C. secundus*, the conclusions probably apply in general to termites that nest in one piece of wood (about 17 % of all described species; [10]) as they share the characteristics of totipotent 'workers' (large immatures) that do not engage in costly foraging behaviour (reviewed in [7]). As such wood-nesting species are supposed to present the ancestral state in termite evolution (reviewed in [7,10]), this opens the possibility that in termites costly altruistic helping by workers evolved as a second step. Preliminary data for *C. secundus *indicate that 'large immatures' stay at the natal nest, which presents a safe heaven with plenty of food, to gain direct fitness by inheriting the breeding position. Thus they resemble philopatric offspring in some vertebrate societies that apparently do little or no work, but wait to become the next breeder (e.g. [18,19]).

## Conclusion

In contrast to general perception where termite workers are considered equivalent to workers in Hymenoptera, the 'large immatures' of *C. secundus *did not behave as workers that help in raising younger siblings. These results, which are to my knowledge the first on the ultimate function of 'workers' in a phylogenetical basal termite, suggest that costly altruistic helping in termites only evolved after staying for direct benefits had evolved. Such altruistic castes are nowadays represented by the soldiers and the 'true workers' of non-wood nesting termites.

## Methods

### Do 'large immatures' stay when the number of young offspring is increased?

In 2003, colonies of *C. secundus *were collected from dead *Ceriops tagal *trees from a mangrove area near Palmerston-Channel Island in Darwin Harbour (Northern Territory, Australia; 12°30' S, 131°0' E) [17]. For the experiment I used twenty-four monogamous field-collected colonies, forming eight triplets each of the same final colony size to control for colony size effects. The relative composition of the original colonies (i.e. the relative proportions of larval instars, large immatures and soldiers) did not differ significantly between the triplets (MANOVA: total: *F*_*6,40 *_= 1.29, *p *= 0.283). Each triplet consisted of one colony that had its natural colony composition (control), one colony where the composition was manipulated by adding around 20 % more young instars (1^st^- and 2^nd^-instar) and eggs than it had originally ('add young'), and one colony where individuals were added, but the age composition was not changed ('add all'). The third trial served as control to check for handling artifacts of adding individuals. The added individuals stemmed from further colonies that had been collected at the same time and place in the field. The colony size and relative composition of the donor colonies did not differ significantly from those of the experimental colonies (control, add young, add all) before the manipulation (colony size: ANOVA: *F*_*3,28 *_= 1.85, *p *= 0.162; relative composition: MANOVA: total: *F*_*9,84 *_= 1.65, *p *= 0.101). Addition of individuals is possible; no aggression occurs and mixing does not affect colony- or individual development [16]. This was further confirmed by this study. The total number of individuals at the end of the experiment did not differ between the three trials (mean ± S.D.: control: 88.7 ± 25.7; add all: 66.1 ± 22.8; add young: 77.6 ± 21.6; ANOVA: *F*_*2,21 *_= 0.23, *p *= 0.794).

Furthermore, no differences were found in the number of 'large immatures' leaving the nest (see Results). Thus, there was no effect of adding individuals that could have masked an effect of having more young to help. The addition occurred at the limited crucial time of the year, after the nuptial flight in August, when developmental 'decisions' about dispersal are reached [12]. Colonies were set up in *Pinus radiata *wood blocks providing abundant food conditions (1 termite : 10 cm^3 ^wood) [17]. The use of *P. radiata *wood does not affect the termites [12,17]. No more than two weeks after the colonies had been collected, they were placed back in the field to the site where they came from. During the set-up of the experimental colonies in the laboratory in Darwin the colonies where kept under conditions similar to the field and appropriate for *C. secundus *[17]. Prior to the swarming period of the next year they were sampled to determine colony composition and the number of dispersing individuals. The latter consisted of winged individuals (alates) as well as last nymphal instars that are known to disperse during the following swarming period [12].

### Data analysis

The number of dispersing individuals were analysed with a one-way ANCOVA using the three trials as factors and colony size as covariate. For non-significant results the effect size index f for an ANCOVA design [20] was calculated using the computer program GPOWER [21]. Additionally, partial eta^2 ^scores were provided as implemented in SPSS to show the effect size of non-significant trial effects relative to other effects (colony size). To compare the total number of individuals at the end of the experiment between control and 'add young' colonies a t-test was applied. The correlation between the number of dispersing 'large immatures' and the number of young in the nest was analysed using Pearson correlation.

### Do 'large immatures' help?

To test whether 'large immatures' help to raise siblings, behavioural observations were performed. Five complete monogamous colonies were collected from the field and were set up in wood blocks with an observation chamber as described elsewhere [22]. Colonies were allowed to resettle for one day. Two 'large immatures' in each colony were marked and focal observations were done six times for 15 minutes during the following three weeks (each individual was observed twice per week; for details see [22]). The following potentially 'altruistic' behaviours were analysed; they constituted the only behaviours that were interactive and that might be altruistic in the sense that they might provide benefits to the recipient at a cost to the donor [22]:

Allogrooming: the individual grooms another individual by moving the mouth parts over the others body.

Proctodeal feeding: the individual feeds another individual by donating substances via the anus; anus-mouth contact.

We distinguished whether an individual actively performed a behaviour or whether it was the passive recipient of a behaviour that had been initiated by another individual.

Additionally, we performed systematic behavioural observations of more than 600 marked individuals from more than 40 colonies during all seasons since this project started in 1999, covering more than 300 observation hours. These observations were done as described above, but individuals were observed only once [16,22].

## Competing interests

The author declares that she has no competing interests.

## Authors' contributions

The author designed, performed and analysed the experiments and wrote the manuscript.
